# Transparent reporting of a multivariable prediction model for individual prognosis or diagnosis (TRIPOD): The TRIPOD statement

**DOI:** 10.1038/bjc.2014.639

**Published:** 2015-01-06

**Authors:** G S Collins, J B Reitsma, D G Altman, K G M Moons

**Affiliations:** 1 Centre for Statistics in Medicine, Nuffield Department of Orthopaedics, Rheumatology and Musculoskeletal Sciences, Botnar Research Centre, University of Oxford, Oxford OX3 7LD, UK; 2 Julius Center for Health Sciences and Primary Care, University Medical Center Utrecht, PO Box 85500, 3508GA Utrecht, The Netherlands

**Keywords:** prediction models, diagnostic, prognostic, model development, model validation, transparent reporting

## Abstract

Prediction models are developed to aid health-care providers in estimating the probability or risk that a specific disease or condition is present (diagnostic models) or that a specific event will occur in the future (prognostic models), to inform their decision making. However, the overwhelming evidence shows that the quality of reporting of prediction model studies is poor. Only with full and clear reporting of information on all aspects of a prediction model can risk of bias and potential usefulness of prediction models be adequately assessed. The Transparent Reporting of a multivariable prediction model for Individual Prognosis Or Diagnosis (TRIPOD) Initiative developed a set of recommendations for the reporting of studies developing, validating, or updating a prediction model, whether for diagnostic or prognostic purposes. This article describes how the TRIPOD Statement was developed. An extensive list of items based on a review of the literature was created, which was reduced after a Web-based survey and revised during a 3-day meeting in June 2011 with methodologists, health-care professionals, and journal editors. The list was refined during several meetings of the steering group and in e-mail discussions with the wider group of TRIPOD contributors. The resulting TRIPOD Statement is a checklist of 22 items, deemed essential for transparent reporting of a prediction model study. The TRIPOD Statement aims to improve the transparency of the reporting of a prediction model study regardless of the study methods used. The TRIPOD Statement is best used in conjunction with the TRIPOD explanation and elaboration document. To aid the editorial process and readers of prediction model studies, it is recommended that authors include a completed checklist in their submission (also available at www.tripod-statement.org).

In medicine, patients with their care providers are confronted with making numerous decisions on the basis of an estimated risk or probability that a specific disease or condition is present (diagnostic setting) or a specific event will occur in the future (prognostic setting) (Box A). In the diagnostic setting, the probability that a particular disease is present can be used, for example, to inform the referral of patients for further testing, initiate treatment directly, or reassure patients that a serious cause for their symptoms is unlikely. In the prognostic setting, predictions can be used for planning lifestyle or therapeutic decisions based on the risk for developing a particular outcome or state of health within a specific period (Steyerberg, 2009; Moons *et al*, 2009b). Such estimates of risk can also be used to risk-stratify participants in therapeutic clinical trials (Hayward *et al*, 2006; Dorresteijn *et al*, 2011).

In both the diagnostic and prognostic setting, estimates of probabilities are rarely based on a single predictor (Riley *et al*, 2013). Doctors naturally integrate several patient characteristics and symptoms (predictors, test results) to make a prediction (see Box B for differences in common terminology between diagnostic and prognostic studies). Prediction is therefore inherently multivariable. Prediction models (also commonly called ‘prognostic models,' ‘risk scores,' or ‘prediction rules' (Steyerberg *et al*, 2013) are tools that combine multiple predictors by assigning relative weights to each predictor to obtain a risk or probability (Steyerberg, 2009; Moons *et al*, 2009b). Well-known prediction models include the Framingham Risk Score (Anderson *et al*, 1991), Ottawa Ankle Rules (Stiell *et al*, 1992), EuroScore (Nashef *et al*, 1999), Nottingham Prognostic Index (Haybittle *et al*, 1982), and the Simplified Acute Physiology Score (Le Gall *et al*, 1984).

## Prediction model studies

**Figure 1:**
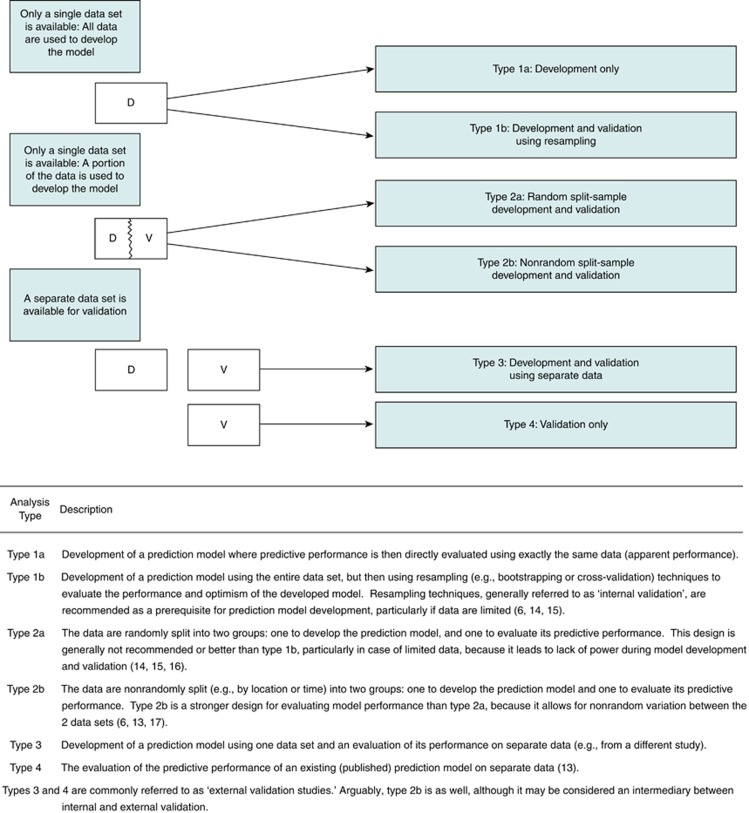
**Types of prediction model studies covered by the TRIPOD statement.** D=development data; V=validation data.

Prediction model studies can be broadly categorised as model development (Royston *et al*, 2009), model validation (with or without updating) (Moons *et al*, 2012a), or a combination of both (Figure 1). Model development studies aim to derive a prediction model by selecting the relevant predictors and combining them statistically into a multivariable model. Logistic and Cox regression are most frequently used for short-term (e.g., disease absent *vs* present, 30-day mortality) and long-term (e.g., 10-year risk) outcomes, respectively (Justice *et al*, 1999; Steyerberg *et al*, 2001b; Altman *et al*, 2009; Royston *et al*, 2009; Moons *et al*, 2012a, 2012b). Studies may also focus on quantifying the incremental or added predictive value of a specific predictor (e.g., newly discovered) to a prediction model (Steyerberg *et al*, 2012).

Quantifying the predictive ability of a model on the same data from which the model was developed (often referred to as apparent performance) will tend to give an optimistic estimate of performance, owing to overfitting (too few outcome events relative to the number of candidate predictors) and the use of predictor selection strategies (Steyerberg *et al*, 2003). Studies developing new prediction models should therefore always include some form of internal validation to quantify any optimism in the predictive performance (e.g., calibration and discrimination) of the developed model. Internal validation techniques use only the original study sample and include such methods as bootstrapping or cross-validation. Internal validation is a necessary part of model development (Steyerberg, 2009). Overfitting, optimism, and miscalibration may also be addressed and accounted for during the model development by applying shrinkage (e.g., heuristic or based on bootstrapping techniques) or penalisation procedures (e.g., ridge regression or lasso) (Steyerberg *et al*, 2001a).

After developing a prediction model, it is strongly recommended to evaluate the performance of the model in other participant data than was used for the model development. Such external validation requires that for each individual in the new data set, outcome predictions are made using the original model (i.e., the published regression formula) and compared with the observed outcomes (Altman *et al*, 2009; Moons *et al*, 2012a). External validation may use participant data collected by the same investigators, typically using the same predictor and outcome definitions and measurements, but sampled from a later period (temporal or narrow validation); by other investigators in another hospital or country, sometimes using different definitions and measurements (geographic or broad validation); in similar participants but from an intentionally different setting (e.g., model developed in secondary care and assessed in similar participants but selected from primary care); or even in other types of participants (e.g., model developed in adults and assessed in children, or developed for predicting fatal events and assessed for predicting nonfatal events) (Justice *et al*, 1999; Reilly and Evans, 2006; Wallace *et al*, 2011; Moons *et al*, 2012a, 2012b). In case of poor performance, the model can be updated or adjusted on the basis of the validation data set (Moons *et al*, 2012a).

## Reporting of multivariable prediction model studies

Studies developing or validating a multivariable prediction model share specific challenges for researchers (Steyerberg *et al*, 2013). Several reviews have evaluated the quality of published reports that describe the development or validation prediction models (Laupacis *et al*, 1997; Mallett *et al*, 2010; Collins *et al*, 2011; Bouwmeester *et al*, 2012; Collins *et al*, 2013, 2014). For example, Mallett *et al* (2010) examined 47 reports published in 2005 presenting new prediction models in cancer. Reporting was found to be poor, with insufficient information described in all aspects of model development, from descriptions of patient data to statistical modeling methods. Collins *et al* (2011) evaluated the methodological conduct and reporting of 39 reports published before May 2011 describing the development of models to predict prevalent or incident type 2 diabetes. Reporting was also found to be generally poor, with key details on which predictors were examined, the handling and reporting of missing data, and model-building strategy often poorly described. Bouwmeester *et al* (2012) evaluated 71 reports, published in 2008 in six high-impact general medical journals, and likewise observed an overwhelmingly poor level of reporting. These and other reviews provide a clear picture that, across different disease areas and different journals, there is a generally poor level of reporting of prediction model studies (Laupacis *et al*, 1997; Ettema *et al*, 2010; Mallett *et al*, 2010; Collins *et al*, 2011; Bouwmeester *et al*, 2012; Collins *et al*, 2013; Steyerberg *et al*, 2013; Collins *et al*, 2014). Furthermore, these reviews have shown that serious deficiencies in the statistical methods, use of small data sets, inappropriate handling of missing data, and lack of validation are common (Laupacis *et al*, 1997; Ettema *et al*, 2010; Mallett *et al*, 2010; Collins *et al*, 2011; Bouwmeester *et al*, 2012; Collins *et al*, 2013; Steyerberg *et al*, 2013; Collins *et al*, 2014). Such deficiencies ultimately lead to prediction models that are not or should not be used. It is therefore not surprising, and fortunate, that very few prediction models, relative to the large number of models published, are widely implemented or used in clinical practice (Steyerberg *et al*, 2013).

Prediction models in medicine have proliferated in recent years. Health-care providers and policy makers are increasingly recommending the use of prediction models within clinical practice guidelines to inform decision making at various stages in the clinical pathway (Rabar *et al*, 2012; Goff *et al*, 2014). It is a general requirement of reporting of research that other researchers can, if required, replicate all the steps taken and obtain the same results (Laine *et al*, 2007). It is therefore essential that key details of how a prediction model was developed and validated be clearly reported to enable synthesis and critical appraisal of all relevant information (Knottnerus, 1995; Altman *et al*, 2009; Collins and Moons, 2012; Seel *et al*, 2012; Siontis *et al*, 2012).

## Reporting guidelines for prediction model studies: The TRIPOD statement

We describe the development of the TRIPOD (Transparent Reporting of a multivariable prediction model for Individual Prognosis or Diagnosis) Statement, a guideline specifically designed for the reporting of studies developing or validating a multivariable prediction model, whether for diagnostic or prognostic purposes. TRIPOD is not intended for multivariable modeling in etiologic studies or for studies investigating single prognostic factors (McShane *et al*, 2005). Furthermore, TRIPOD is also not intended for impact studies that quantify the impact of using a prediction model on participant or doctors' behavior and management, participant health outcomes, or cost-effectiveness of care, compared with not using the model (Moons *et al*, 2009a, 2012a).

Reporting guidelines for observational (the STrengthening the Reporting of OBservational studies in Epidemiology (STROBE)) (von Elm *et al*, 2007), tumor marker (REporting recommendations for tumour MARKer prognostic studies (REMARK)) (McShane *et al*, 2005), diagnostic accuracy (STAndards for the Reporting of Diagnostic accuracy studies (STARD)) (Bossuyt *et al*, 2003), and genetic risk prediction (Genetic RIsk Prediction Studies (GRIPS)) (Janssens *et al*, 2011b) studies all contain many items that are relevant to studies developing or validating prediction models. However, none of these guidelines are entirely appropriate for prediction model studies. The two guidelines most closely related to prediction models are REMARK and GRIPS. However, the focus of the REMARK checklist is primarily on prognostic factors and not prediction models, whereas the GRIPS statement is aimed at risk prediction using genetic risk factors and the specific methodological issues around handling large numbers of genetic variants.

To address a broader range of studies, we developed the TRIPOD guideline: Transparent Reporting of a multivariable prediction model for Individual Prognosis or Diagnosis. TRIPOD explicitly covers the development and validation of prediction models for both diagnosis and prognosis, for all medical domains and all types of predictors. TRIPOD also places much more emphasis on validation studies and the reporting requirements for such studies. The reporting of studies evaluating the incremental value of specific predictors, beyond established predictors or even beyond existing prediction models (Tzoulaki *et al*, 2011; Steyerberg *et al*, 2012), also fits entirely within the remit of TRIPOD (see the accompanying explanation and elaboration document (Moons *et al*, 2015), available at www.annals.org).

## Developing the TRIPOD statement

We convened a 3-day meeting with an international group of prediction model researchers, including statisticians, epidemiologists, methodologists, health-care professionals, and journal editors (from *Annals of Internal Medicine*, *BMJ*, *Journal of Clinical Epidemiology,* and *PLoS Medicine*) to develop recommendations for the TRIPOD Statement.

We followed published guidance for developing reporting guidelines and established a steering committee (Drs Collins, Reitsma, Altman, and Moons) to organise and coordinate the development of TRIPOD (Moher *et al*, 2010). We conducted a systematic search of MEDLINE, EMBASE, PsychINFO, and Web of Science to identify any published articles making recommendations on reporting of multivariable prediction models (or aspects of developing or validating a prediction model), reviews of published reports of multivariable prediction models that evaluated methodological conduct or reporting and reviews of methodological conduct and reporting of multivariable models in general. From these studies, a list of 129 possible checklist items was generated. The steering committee then merged related items to create a list of 76 candidate items.

Twenty-five experts with a specific interest in prediction models were invited by e-mail to participate in the Web-based survey and to rate the importance of the 76 candidate checklist items. Respondents (24 of 27) included methodologists, health-care professionals, and journal editors. (In addition to the 25 meeting participants, the survey was also completed by 2 statistical editors from *Annals of Internal Medicine*.)

The results of the survey were presented at a 3-day meeting in June 2011, in Oxford, United Kingdom; it was attended by 24 of the 25 invited participants (22 of whom had participated in the survey). During the 3-day meeting, each of the 76 candidate checklist items was discussed in turn, and a consensus was reached on whether to retain, merge with another item, or omit the item. Meeting participants were also asked to suggest additional items. After the meeting, the checklist was revised by the steering committee during numerous face-to-face meetings, and circulated to the participants to ensure it reflected the discussions. While making revisions, conscious efforts were made to harmonise our recommendations with other reporting guidelines, and where possible we chose the same or similar wording for items (McShane *et al*, 2005; von Elm *et al*, 2007; Little *et al*, 2009; Moher *et al*, 2009; Janssens *et al*, 2011b).

## TRIPOD Components

The TRIPOD Statement is a checklist of 22 items that we consider essential for good reporting of studies developing or validating multivariable prediction models (Table 1). The items relate to the title and abstract (items 1 and 2), background and objectives (item 3), methods (items 4 through 12), results (items 13 through 17), discussion (items 18 through 20), and other information (items 21 and 22). The TRIPOD Statement covers studies that report solely development (Royston *et al*, 2009, Moons *et al*, 2012b), both development and external validation, and solely external validation (with or without updating), of a prediction model (Altman *et al*, 2009) (Figure 1). Therefore, some items are relevant only for studies reporting the development of a prediction model (items 10a, 10b, 14, and 15), and others apply only to studies reporting the (external) validation of a prediction model (items 10c, 10e, 12, 13c, 17, and 19a). All other items are relevant to all types of prediction model development and validation studies. Items relevant only to the development of a prediction model are denoted by *D*, items relating solely to a validation of a prediction model are denoted by *V*, whereas items relating to both types of study are denoted *D;V*.

The recommendations within TRIPOD are guidelines only for reporting research and do not prescribe how to develop or validate a prediction model. Furthermore, the checklist is not a quality assessment tool to gauge the quality of a multivariable prediction model.

An ever-increasing number of studies are evaluating the incremental value of specific predictors, beyond established predictors or even beyond existing prediction models (Tzoulaki *et al*, 2011; Steyerberg *et al*, 2012). The reporting of these studies fits entirely within the remit of TRIPOD (see accompanying explanation and elaboration document (Moons *et al*, 2015).

## The TRIPOD explanation and elaboration document

In addition to the TRIPOD Statement, we produced a supporting explanation and elaboration document (Moons *et al*, 2015) in a similar style to those for other reporting guidelines (Vandenbroucke *et al*, 2007; Janssens *et al*, 2011a; Altman *et al*, 2012). Each checklist item is explained and accompanied by examples of good reporting from published articles. In addition, because many such studies are methodologically weak, we also summarise the qualities of good (and the limitations of less good) studies, regardless of reporting (Moons *et al*, 2015). A comprehensive evidence base from existing systematic reviews of prediction models was used to support and justify the rationale for including and illustrating each checklist item. The development of the explanation and elaboration document was completed after several face-to-face meetings, teleconferences, and iterations among the authors. Additional revisions were made after sharing the document with the whole TRIPOD group before final approval.

## Role of the funding source

There was no explicit funding for the development of this checklist and guidance document. The consensus meeting in June 2011 was partially funded by a National Institute for Health Research Senior Investigator Award held by Dr Altman, Cancer Research UK, and the Netherlands Organization for Scientific Research. Drs Collins and Altman are funded in part by the Medical Research Council. Dr Altman is a member of the Medical Research Council Prognosis Research Strategy (PROGRESS) Partnership. The funding sources had no role in the study design, data collection, analysis, preparation of the manuscript, or decision to submit the manuscript for publication.

## Discussion

Many reviews have showed that the quality of reporting in published articles describing the development or validation of multivariable prediction models in medicine is poor (Laupacis *et al*, 1997; Ettema *et al*, 2010; Mallett *et al*, 2010; Collins *et al*, 2011; Bouwmeester *et al*, 2012; Collins *et al*, 2013, 2014). In the absence of detailed and transparent reporting of the key study details, it is difficult for the scientific and health-care community to objectively judge the strengths and weaknesses of a prediction model study (Collins and Michaëlsson, 2012; Seel *et al*, 2012; Järvinen *et al*, 2014). The explicit aim of this checklist is to improve the quality of reporting of published prediction model studies. The TRIPOD guideline has been developed to support authors in writing reports describing the development, validation, or updating of prediction models, aid editors and peer reviewers in reviewing manuscripts submitted for publication, and help readers in critically appraising published reports.

The TRIPOD Statement does not prescribe how studies developing, validating, or updating prediction models should be undertaken, nor should it be used as a tool for explicitly assessing quality or quantifying risk of bias in such studies (Moons *et al*, 2014). There is, however, an implicit expectation that authors have an appropriate study design and conducted certain analyses to ensure all aspects of model development and validation are reported. The accompanying explanation and elaboration document describes aspects of good practice for such studies, as well as highlighting some inappropriate approaches that should be avoided (Moons *et al*, 2015).

TRIPOD encourages complete and transparent reporting reflecting study design and conduct. It is a minimum set of information that authors should report to inform the reader about how the study was carried out. We are not suggesting a standardised structure of reporting, rather that authors should ensure that they address all the checklist items somewhere in their article with sufficient detail and clarity.

We encourage researchers to develop a study protocol, especially for model development studies, and even register their study in registers that accommodate observational studies (such as ClinicalTrials.gov) (Hemingway *et al*, 2009; Williams *et al*, 2010). The importance of also publishing protocols for developing or validating prediction models, certainly when conducting a prospective study, is slowly being acknowledged (Stiell *et al*, 2002a, 2002b). Authors can also include the study protocol when submitting their article for peer review, so that readers can know the rationale for including individuals into the study or whether all of the analyses were prespecified.

To help the editorial process, peer reviewers, and, ultimately, readers, we recommend submitting the checklist as an additional file with the report, indicating the pages where information for each item is reported. The TRIPOD reporting template for the checklist can be downloaded from www.tripod-statement.org.

Announcements and information relating to TRIPOD will be broadcasted on the TRIPOD Twitter address (@TRIPODStatement). The Enhancing the QUAlity and Transparency Of health Research (EQUATOR) Network (www.equator-network.org) will help disseminate and promote the TRIPOD Statement.

Methodological issues in developing, validating, and updating prediction models evolve. TRIPOD will be periodically reappraised, and if necessary, modified to reflect comments, criticisms, and any new evidence. We therefore encourage readers to make suggestions for future updates so that ultimately, the quality of prediction model studies will improve.

## Figures and Tables

**Table 1:** Checklist of items to include when reporting a study developing or validating a multivariable prediction model for diagnosis or prognosis^a^

**Section/topic**	**Item**	**Development or validation?**	**Checklist item**	**Page**
**Title and abstract**				
Title	1	D;V	Identify the study as developing and/or validating a multivariable prediction model, the target population, and the outcome to be predicted.	
Abstract	2	D;V	Provide a summary of objectives, study design, setting, participants, sample size, predictors, outcome, statistical analysis, results, and conclusions.	
**Introduction**				
Background and objectives	3a	D;V	Explain the medical context (including whether diagnostic or prognostic) and rationale for developing or validating the multivariable prediction model, including references to existing models.	
	3b	D;V	Specify the objectives, including whether the study describes the development or validation of the model, or both.	
**Methods**				
Source of data	4a	D;V	Describe the study design or source of data (e.g., randomised trial, cohort, or registry data), separately for the development and validation data sets, if applicable.	
	4b	D;V	Specify the key study dates, including start of accrual; end of accrual; and, if applicable, end of follow-up.	
Participants	5a	D;V	Specify key elements of the study setting (e.g., primary care, secondary care, general population) including number and location of centres.	
	5b	D;V	Describe eligibility criteria for participants.	
	5c	D;V	Give details of treatments received, if relevant.	
Outcome	6a	D;V	Clearly define the outcome that is predicted by the prediction model, including how and when assessed.	
	6b	D;V	Report any actions to blind assessment of the outcome to be predicted.	
Predictors	7a	D;V	Clearly define all predictors used in developing the multivariable prediction model, including how and when they were measured.	
	7b	D;V	Report any actions to blind assessment of predictors for the outcome and other predictors.	
Sample size	8	D;V	Explain how the study size was arrived at.	
Missing data	9	D;V	Describe how missing data were handled (e.g., complete-case analysis, single imputation, multiple imputation) with details of any imputation method.	
Statistical analysis methods	10a	D	Describe how predictors were handled in the analyses.	
	10b	D	Specify type of model, all model-building procedures (including any predictor selection), and method for internal validation.	
	10c	V	For validation, describe how the predictions were calculated.	
	10d	D;V	Specify all measures used to assess model performance and, if relevant, to compare multiple models.	
	10e	V	Describe any model updating (e.g., recalibration) arising from the validation, if done.	
Risk groups	11	D;V	Provide details on how risk groups were created, if done.	
Development *vs* validation	12	V	For validation, identify any differences from the development data in setting, eligibility criteria, outcome, and predictors.	
**Results**				
Participants	13a	D;V	Describe the flow of participants through the study, including the number of participants with and without the outcome and, if applicable, a summary of the follow-up time. A diagram may be helpful.	
	13b	D;V	Describe the characteristics of the participants (basic demographics, clinical features, available predictors), including the number of participants with missing data for predictors and outcome.	
	13c	V	For validation, show a comparison with the development data of the distribution of important variables (demographics, predictors, and outcome).	
Model development	14a	D	Specify the number of participants and outcome events in each analysis.	
	14b	D	If done, report the unadjusted association between each candidate predictor and outcome.	
Model specification	15a	D	Present the full prediction model to allow predictions for individuals (i.e., all regression coefficients, and model intercept or baseline survival at a given time point).	
	15b	D	Explain how to use the prediction model.	
Model performance	16	D;V	Report performance measures (with CIs) for the prediction model.	
Model updating	17	V	If done, report the results from any model updating (i.e., model specification, model performance).	
**Discussion**				
Limitations	18	D;V	Discuss any limitations of the study (such as nonrepresentative sample, few events per predictor, missing data).	
Interpretation	19a	V	For validation, discuss the results with reference to performance in the development data, and any other validation data.	
	19b	D;V	Give an overall interpretation of the results, considering objectives, limitations, results from similar studies, and other relevant evidence.	
Implications	20	D;V	Discuss the potential clinical use of the model and implications for future research	
**Other information**				
Supplementary information	21	D;V	Provide information about the availability of supplementary resources, such as study protocol, Web calculator, and data sets.	
Funding	22	D;V	Give the source of funding and the role of the funders for the present study.	

a Items relevant only to the development of a prediction model are denoted by *D*, items relating solely to a validation of a prediction model are denoted by *V*, and items relating to both are denoted *D;V*. We recommend using the TRIPOD Checklist in conjunction with the TRIPOD explanation and elaboration document.

## Box A. Schematic representation of diagnostic and prognostic prediction modeling studies

The nature of the prediction in diagnosis is estimating the probability that a specific outcome or disease is present (or absent) within an individual, at this point in time—that is, the moment of prediction (T=0). In prognosis, the prediction is about whether an individual will experience a specific event or outcome within a certain time period. In other words, in diagnostic prediction, the interest is in principle a cross-sectional relationship, whereas prognostic prediction involves a longitudinal relationship. Nevertheless, in diagnostic modeling studies, for logistical reasons, a time window between predictor (index test) measurement and the reference standard is often necessary. Ideally, this interval should be as short as possible and without starting any treatment within this period.

## Appendix 1

### Appendix 1 Members of the TRIPOD Group

Gary Collins (University of Oxford, Oxford, United Kingdom); Douglas Altman (University of Oxford, Oxford, United Kingdom); Karel Moons (University Medical Center Utrecht, Utrecht, The Netherlands); Johannes Reitsma (University Medical Center Utrecht, Utrecht, the Netherlands); Virginia Barbour (*PLoS Medicine*, United Kingdom and Australia); Nancy Cook (Division of Preventive Medicine, Brigham & Women's Hospital, Boston, Massachusetts); Joris de Groot (University Medical Center Utrecht, Utrecht, The Netherlands); Trish Groves (*BMJ*, London, United Kingdom); Frank Harrell, Jr. (Vanderbilt University, Nashville, Tennessee); Harry Hemingway (University College London, London, United Kingdom); John Ioannidis (Stanford University, Stanford, California); Michael W. Kattan (Cleveland Clinic, Cleveland, Ohio); André Knottnerus (Maastricht University, Maastricht, The Netherlands, and *Journal of Clinical Epidemiology*); Petra Macaskill (University of Sydney, Sydney, Australia); Susan Mallett (University of Oxford, Oxford, United Kingdom); Cynthia Mulrow (*Annals of Internal Medicine*, American College of Physicians, Philadelphia, Pennsylvania); David Ransohoff (University of North Carolina at Chapel Hill, Chapel Hill, North Carolina); Richard Riley (University of Birmingham, Birmingham, United Kingdom); Peter Rothwell (University of Oxford, Oxford, United Kingdom); Patrick Royston (Medical Research Council Clinical Trials Unit at University College London, London, United Kingdom); Willi Sauerbrei (University of Freiburg, Freiburg, Germany); Ewout Steyerberg (University Medical Center Rotterdam, Rotterdam, the Netherlands); Ian Stiell (University of Ottawa, Ottawa, Ontario, Canada); Andrew Vickers (Memorial Sloan Kettering Cancer Center, New York).
